# Oviposition in the blood-sucking insect *Rhodnius prolixus* is modulated by host odors

**DOI:** 10.1186/s13071-015-0867-5

**Published:** 2015-05-09

**Authors:** Fabio Guidobaldi, Pablo G Guerenstein

**Affiliations:** Laboratorio de Neuroetología Ecológica, CICyTTP-CONICET, Diamante, Entre Ríos Argentina; Facultad de Ingeniería, Universidad Nacional de Entre Ríos, Oro Verde, Entre Ríos Argentina

**Keywords:** Oviposition stimulation, Spatial distribution of eggs, Chagas disease, Triatomine, Hematophagous insect, Insect control, Insect monitoring

## Abstract

**Background:**

Triatomine bugs are blood-sucking insects, vectors of Chagas disease. Despite their importance, their oviposition behavior has received relatively little attention. Some triatomines including *Rhodnius prolixus* stick their eggs to a substrate. It is known that mechanical cues stimulate oviposition in this species*.* However, it is not clear if chemical signals play a role in this behavior. We studied the role of host cues, including host odor, in the oviposition behavior of the triatomine *R. prolixus*.

**Methods:**

Tests were carried out in an experimental arena and stimuli consisted of a mouse or hen feathers. The number of eggs laid and the position of those eggs with respect to the stimulus source were recorded. Data were analyzed using the Mann-Whitney and Kruskal-Wallis tests.

**Results:**

Both a mouse and hen feathers stimulated oviposition. In addition, hen feathers evoked a particular spatial distribution of eggs that was not observed in the case of mouse.

**Conclusions:**

We propose that volatile chemical cues from the host play a role in the oviposition behavior of triatomines that stick their eggs. Thus, host odor would stimulate and spatially guide oviposition.

**Electronic supplementary material:**

The online version of this article (doi:10.1186/s13071-015-0867-5) contains supplementary material, which is available to authorized users.

## Background

The selection of sites for oviposition is a critical factor for the survival of the offspring of an individual. It has been suggested that the oviposition behavior of phytophagous insects plays an important role in host specificity, the origins of host shifts, sympatric speciation and co-evolution [1]. Moreover, offspring survival is one of the most important components of lifetime reproductive success [2]. In organisms with rudimentary forms of parental care or with no parental care, oviposition site selection should have a considerable impact on parental fitness since it often determines to a great extent the chances of survival of the offspring [3].

Thus, females of phytophagous and parasitoid insects place their eggs in sites where their offspring will find abundant and high-quality food resources, and/or enemy-free sites [4-9].

In those cases in which optimal oviposition sites are rare or difficult to find, female’s fitness would increase if oviposition is stimulated upon an encounter with a preferred site. Strong evidence suggests that ovarian dynamics in insects respond to variability in host quality and availability in adaptive ways [8].

Among blood-sucking insects, mosquitoes may be attracted or repelled by environmental cues, modulating the search for an oviposition site [10,11]. Thus, several studies have reported a role of identified oviposition site semiochemicals, like those originating from grass infusions, as orientation cues and/or stimulators of egg-laying behavior in mosquitoes [12-19]. Moreover, pheromones stimulating oviposition were also identified (e.g., [13,15]).

Phlebotominae sandflies oviposit in damp, organically rich terrestrial habitats. Gravid females of *Lutzomyia longipalpis* lay their eggs singly though aggregated. It has been shown that an attractive oviposition pheromone is secreted onto the eggs during oviposition, although other cues originating in the oviposition sites would also play a role in attracting females [20].

Triatomine bugs are hematophagous insects, vectors of *Trypanosoma cruzi*, the etiological agent of Chagas Disease, an illness that seriously affects public health throughout Latin America. Vector control is the most effective method to prevent Chagas disease [21]. The oviposition behavior of triatomines has received relatively little attention and it is not clear if chemical signals play a role in their oviposition behavior. Triatomines lay their eggs either free or attached to a substrate, depending on the species. *Rhodnius prolixus*, one of the main vectors of Chagas disease, is a species with arboreal habit, associated with palm trees [22,23], and believed to be associated to birds [22-25]. This species has been observed to stick its eggs to bird’s feathers [22,25]. Thus, for this species the selection of a suitable oviposition site would be related to the availability of a food source for the larvae immediately after hatching. Schilman et al. [25] have shown that in *R. prolixus* feathers induce higher oviposition than a cardboard substrate. The differences in the responses to those two substrates could be due to mechanical, gustatory, or olfactory cues or a combination of them. However, the fact that no statistical differences were found between new feathers (presumably rich in chemical cues) and old feathers (presumably low in chemical cues) did not allow confirmation of a role of chemical cues, although the data showed a tendency for a gustatory/olfactory effect [25]. One of the main obstacles for the triatomine control programs is house re-infestation. Because the oviposition behavior (e.g. number of eggs laid) should have an impact on the growth of recently established populations we investigated the role of host cues, including host odor, in the oviposition behavior of the triatomine *R. prolixus.*

## Methods

### Insects

*R. prolixus* bugs were reared in the laboratory at 27 ± 1°C, ambient RH and a 12:12 artificial light cycle. The insects were provided by the National Chagas Institute of Argentina. They originated from Colombia and were reared for ca. 25 generations in the laboratory. Bugs were fed every 20-30 days on hens. After molting to the adult stage and until the experiments, males and females were kept together in crystal polystyrene recipients with paper as substrate. Experimental insects were chosen at random from a pool of 200-300 of those adults, 3 to 10 days after feeding.

### First and second experimental series

**Figure 1:**
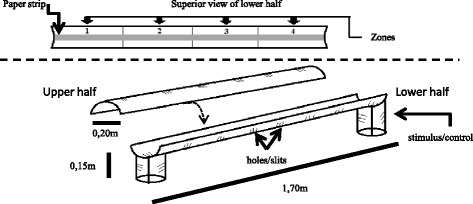
Experimental arena to evaluate the number of eggs laid and oviposition site preferences in *R. prolixus*.

The experimental device consisted of a PVC cylinder (0.2 m diameter, 1.70 m length). This cylinder was held 15 cm above the floor by two PVC columns at each end of it (Figure 1, Additional file 1: Figure S1). The cylinder was longitudinally divided in two halves. Removing the upper half provided easy access to the experimental area during experiment set up. Before the start of an experiment this upper half was placed back on top of the lower half thus sealing the cylinder longitudinally, while the open sides of the cylinder were covered with tissue mesh.

Every 0.2 m, a series of holes (0.5 cm diameter) and slits (0.3 × 5 cm) in the upper and lower halves of the cylinder allowed air circulation. They were covered with a fine tissue mesh to avoid the escape of bugs or eggs. The two columns also had a series of holes and slits. A piece of paper (3 cm width) was placed all along the length of the lower half of the cylinder to provide an optimal substrate on which the bugs could walk (it should be noted that *R. prolixus* adults were also able to walk outside the paper strip, all around the cylinder). The cylinder had waterproof marks to establish four equal-length zones (Figure 1).

Before a test started, six experimental adults (4 females and 2 males) *R. prolixus* were carefully placed on the experimental arena, after which the cylinder was assembled. All the experimental insects originated from a pool of adults of different ages. Thus, the insects used in both the test and control experiments consisted on subpopulations of that pool of adults. The age of individual insects was not controlled. In the test cylinder one of the columns contained the test stimulus while the other one was a control. The position of the test and control columns was switched in successive trials. A control cylinder contained no stimulus at any of the two columns. A series of holes on the base of the cylinder allowed the stimulus to reach the arena from the columns. The test and control cylinders were run simultaneously in separate contiguous rooms.

The experimental rooms were at 27 ± 2°C and had a natural light cycle provided by a partially covered window that allowed dim light during the day. Experiments started at 6 pm and lasted three days, after which the insects were recovered, the eggs in each section of the cylinder were counted and collected, the paper substrate was discarded and the device was thoroughly washed.

In the first experimental series the test stimulus was a live mouse (Balb-C strain) while in the second series the test stimulus consisted of 40 hen feathers carefully collected right before the beginning of the tests. Insects did not have physical access to the stimulus sources.

### Statistical analysis

We established an *a priori* requirement to include an assay in the analysis: only tests with at least 10 eggs in at least one of the cylinders were taken into account for analysis. To address the effect of the stimulus on the site where the eggs were laid (e.g., if the insects prefer to oviposit near the stimulus source), the distribution of the eggs for control and test cylinders was analyzed using the Kruskal-Wallis test. This non parametric test was used as those data did not follow a normal distribution according to the Shapiro-Wilk test (p < 0.05; [26]). In case of a statistically significant effect in the distribution of the eggs, all pair-wise comparisons between zones were analyzed *a posteriori* using the Mann-Whitney test [27]. The external and internal zones of the cylinders provided different physical cues for the insects (Figure 1). Thus, for example, the two external zones (one and four) had tissue mesh on one side while the internal zones (two and three) did not. Therefore, we asked if in the absence of host cues the number of eggs in the external zones of the cylinders was different from that in the internal zones. To study this, data from control cylinders of both mouse and feathers experiments were used and analyzed using the Mann-Whitney test. To analyze if host cues stimulated oviposition, the egg counts in the test cylinders were compared to those in the control cylinders using the Mann-Whitney test.

### Ethical approval

Animals have been treated in accordance with the guidelines of the Comité Institucional para el Cuidado y Uso de Animales de Laboratorio (CICUAL; Institutional Committee for the Care and Use of Laboratory Animals), which are based on the guidelines from the Council for International Organizations of Medical Sciences (CIOMS).

## Results

### First experimental series

**Figure 2:**
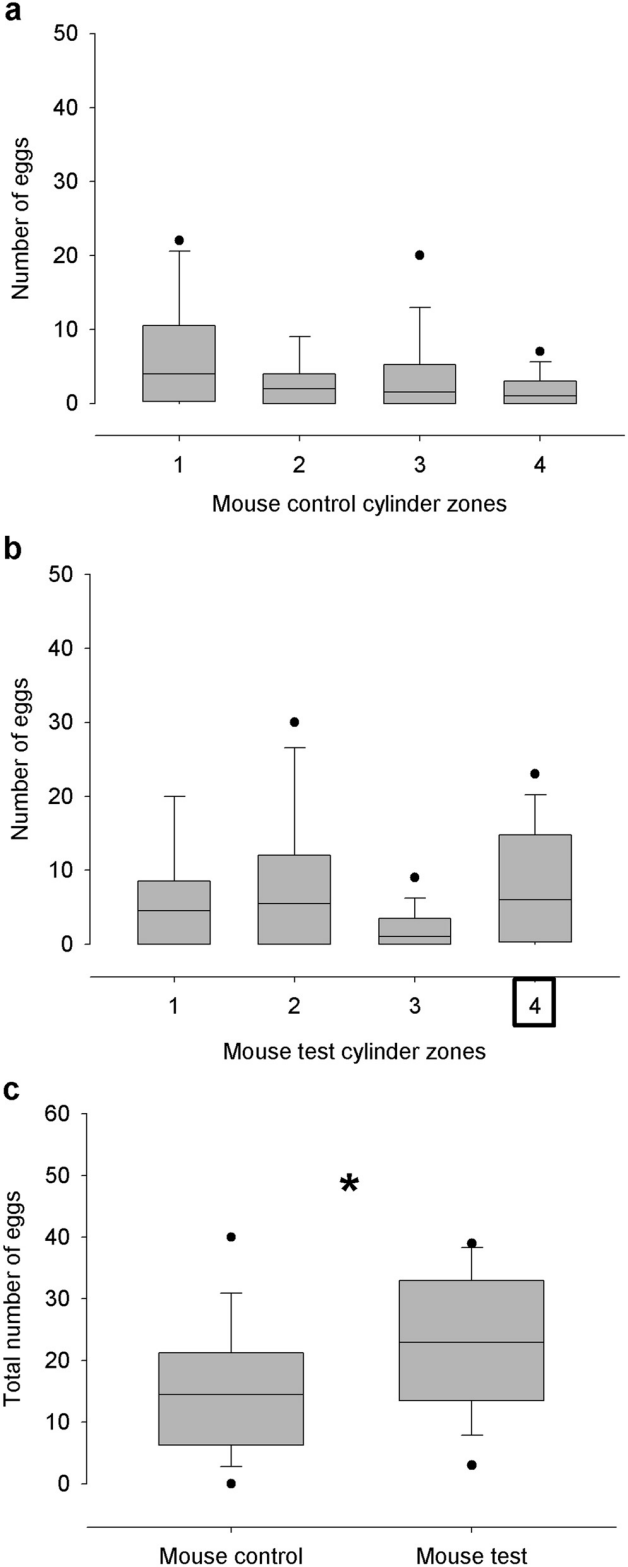
Spatial distribution of eggs and number of eggs laid by *R. prolixus* when the stimulus consisted on a mouse. **a**. Number of eggs at each zone of the control tube. **b**. Number of eggs at each zone of the test tube. Square surrounding number four indicates the position of the stimulus. **c**. Total number of eggs at the control and test tubes. “*”denotes statistical significance (p < 0.05); N = 16 for all figures.

Sixteen out of 32 assays were analyzed (see section Statistical analysis). In the case of the control cylinders, the statistical analysis showed no significant differences in the distribution of eggs between zones (Kruskal-Wallis test, p > 0.05, N = 16, Figure 2a). Similarly, no significant differences in the distribution of eggs between zones for the test cylinders was found (Kruskal-Wallis test, p > 0.05, N = 16, Figure 2b). A mouse stimulated oviposition as the number of eggs in the test cylinders was significantly higher than in the control (369 and 236, respectively; Mann-Whitney test, p < 0.05, N = 16, Figure 2c).

### Second experimental series

**Figure 3:**
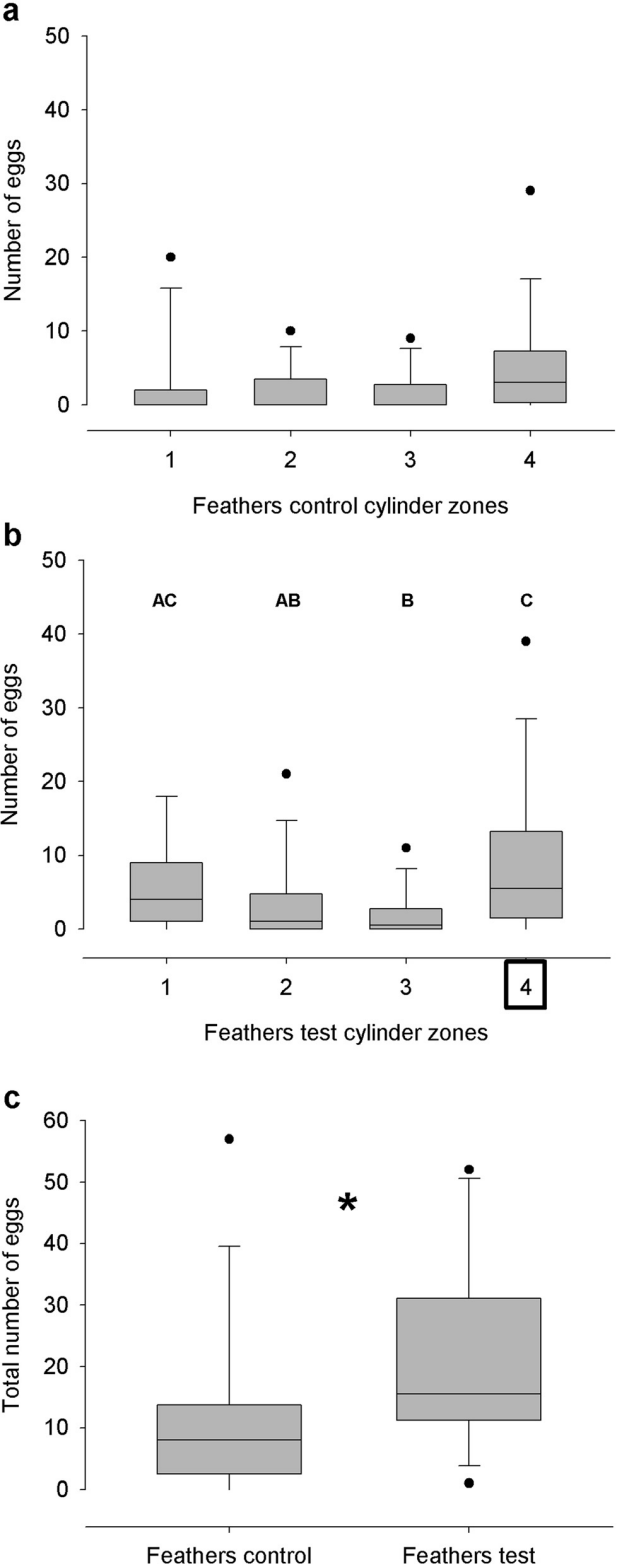
Spatial distribution of eggs and number of eggs laid by *R. prolixus* when the stimulus consisted on hen feathers. **a**. Number of eggs at each zone of the control tube. **b**. Number of eggs at each zone of the test tube. Statistical differences were found between zones. The results of all pair-wise comparisons between zones are shown with letters. Square surrounding number four indicates the position of the stimulus. **c**. Total number of eggs at the control and test tubes. “*”denotes statistical significance (p < 0.05); N = 16 for all figures.

Sixteen out of 40 assays were analyzed (see section Statistical analysis). In the case of the control cylinders, the Kruskal-Wallis test showed no significant differences in the distribution of eggs between zones (p > 0.05, N = 16, Figure 3a). On the contrary, a significant difference in the distribution of eggs between zones was found for the test cylinders (Kruskal-Wallis test p < 0.05, N = 16). The results of all pair-wise comparisons between zones using the Mann-Whitney test are shown in Figure 3b. Thus, the number of eggs in the zone where the feathers were placed was significantly higher than that in the internal zones (p < 0.05), although it was not different from the number of eggs in the opposite end of the cylinder (p > 0.05). However, no significant differences were found in the number of eggs in the external zones with respect to the internal zones in the case of the control cylinder (Mann-Whitney test, p > 0.05, N = 16). Hen feathers stimulated oviposition as the number of eggs in the test cylinder was significantly higher than in the control (332 and 188, respectively; Mann-Whitney test, p < 0.05, N = 16, Figure 3c).

## Discussion

Previous work suggested that a feather substrate induced female’s *R. prolixus* to lay more eggs than on a piece of cardboard [25]. This suggests that oviposition is modulated by mechanical, gustatory, or olfactory cues or a combination of them. However the sensory modality involved could not be teased apart since a comparison between feathers with different levels of chemical cues resulted in no statistical differences. We designed experiments to test the effect of host cues, including host odor, in the oviposition behavior of triatomines. Thus, we asked if those cues could modulate the spatial pattern of oviposition and/or the number of laid eggs.

We found that both a live mouse and hen feathers stimulate oviposition in *R. prolixus* even when the insects could not contact the source of stimulus. It should be noted that the cues emitted from a mouse include heat, water vapor, CO_2_ and odors, and any of them (or a combination of them) could be responsible for the stimulatory effect found. However, it is unlikely that the feathers emitted cues other than odors during the experiments. The simplest interpretation of our results is that host odors alone can stimulate oviposition in *R. prolixus*. However, a role of other host cues cannot be excluded. It should be interesting to study if the odor of a mouse *per se* could also evoke the response obtained in the case of hen feathers.

In addition, in the presence of hen feathers the spatial distribution of eggs was different from that found in the negative control while a live mouse did not evoke such a response.

Thus, we suggest that hen feather odor modulates the spatial distribution of laid eggs. It should be noted that in our experiments we were just interested in the population behavior of the bugs. If we had tested the insects individually, the results might have been different.

Because we cannot directly compare the response to hen feathers to that to a live mouse, it remains unclear if that stimulus-specific response could be related to the natural history of *R. prolixus* as it is believed that the natural hosts of *R. prolixus* are birds. The response obtained could also be due to the fact that our insect colony is fed on hens. In any case, the high number of eggs in the stimulus zone could be related to an attempt to lay eggs close to the host although it is unclear why we also found a high number of eggs at the opposite end of the cylinder. The fact that the spatial distribution of eggs was not modulated by a live mouse suggests that CO_2_ is not involved in this response. In preliminary previous dual-choice experiments using just high CO_2_ as stimulus (1000 ppm and 470 ppm CO_2_, test and control values, respectively) the number of eggs laid on the test and control side was the same (68 eggs, N = 12 assays). This again suggests no role of CO_2_ in the spatial pattern of oviposition.

It should be noted that even when the feathers used as stimuli in the second experimental series were not replaced during the three-day tests, they were still able to modulate significantly both the spatial pattern of oviposition and the number of eggs laid. Moreover, as in our experiments, the insects did not have physical access to the feathers; the responses to this stimulus were not mediated by mechanical nor gustatory stimuli. However gustatory or mechanical stimuli could play a role in the oviposition behavior and could help the insects pin point the optimal oviposition site.

The original and simple experimental design used in the experimental series proved to be useful to study oviposition behavior in triatomines. Our results not only contribute basic knowledge on the chemical ecology of triatomines, but could also be useful to develop a method for vector control. Thus, a host-based synthetic odor blend could be used to lure the insects to oviposit in artificial devices to monitor the presence of the bugs and/or to diminish their populations. This endeavor may need providing the insects mechanical and/or gustatory cues in addition to olfactory ones. In mosquitoes, several oviposition attractants have been proposed (e.g., [28]) that could be used in the popular mosquito “ovitraps”. On the other hand, knowledge on the particular odor constituents that are responsible for the responses observed in this work could be possibly used to develop odor-based strategies to affect negatively the oviposition rate by altering the proportion/s of key constituent/s of host odor turning it inappropriate or by interfering with the receptors of key odor constituents. Those strategies are already being used in the case of the mosquito host finding behavior (e.g., [29,30]).

## Conclusions

We propose that volatile chemical cues from the host play an important role in the oviposition behavior of triatomines. Thus, host odor would stimulate and spatially guide oviposition. Thus, monitoring and control strategies could include odor-based manipulation of the oviposition behavior of triatomines.

## Additional file

Additional file 1: Figure S1. Superior view of the experimental arena used. The photo shows groups of nine holes and four slits distributed symmetrically along the arena to ensure air circulation. The photo also shows a group of four slits that allowed odor stimuli to reach the arena from the stimuli columns. The inset shows a group of holes and a group of slits distributed symmetrically.

## References

[1] Thompson JN, Pellmyr O (1991). Evolution of oviposition behavior and host preference in lepidoptera. Annu Rev Entomol.

[2] Clutton-Brock TH (1988). Reproductive success.

[3] García-Gonzalez F, Gomendio M (2003). Oviposition site selection and oviposition stimulation by conspecifics in the golden egg bug (*Phyllomorpha laciniata*): implications for female fitness. Behav Ecol Sociobiol.

[4] Jaenike J (1978). On optimal oviposition behavior in phytophagous insects. Theor Popul Biol.

[5] Jaenike J (1990). Host specialization in phytophagous insects. Annu Rev Ecol Syst.

[6] Mangel M (1989). An evolutionary interpretation of the “motivation to oviposit”. J Evol Biol.

[7] Godfray HCJ (1994). Parasitoids: behavioral and evolutionary ecology.

[8] Papaj DR (2000). Ovarian dynamics and host use. Annu Rev Entomol.

[9] Papaj DR, Messing RH (1996). Functional shifts in the use of parasitized host by a tephritid fly: the role of host quality. Behav Ecol.

[10] Navarro-Silva MA, Márques FA, Duque LJE (2009). Review of semiochemicals that mediate the oviposition of mosquitoes: a possible sustainable tool for the control and monitoring of *Culicidae*. Revista Brasileira de Entomologia.

[11] Takken W, Knols BGJ (1999). Odor-mediated behavior of afrotropical malaria mosquitoes. Ann Rev Entomol.

[12] Albeny-Simões D, Murrel EG, Elliot SL, Andrade MR, Lima E, Juliano SA, Vilela EF (2014). Attracted to the enemy: *Aedes aegypti* prefers oviposition sites with predator-killed conspecifics. Oecologia.

[13] Olagbemiro TO, Birkett MA, Mordue (Luntz) AJ, Pickett JA (2004). Laboratory and field responses of the mosquito, *Culex quinquefasciatus*, to plant-derived *Culex* spp. Oviposition pheromone and the oviposition cue skatole. J Chem Ecol.

[14] Barbosa RMR, Furtado A, Regis L, Leal WS (2010). Evaluation of an oviposition-stimulating kairomone for the yellow fever mosquito, *Aedes aegypti*, in Recife. Brazil J Vector Ecol.

[15] Pickett JA, Woodcock CM (1996). The role of mosquito olfaction in oviposition site location and in the avoidance of unsuitable hosts. Ciba Found Symp.

[16] Du Y-J, Millar JG (1999). Electroantennogram and oviposition bioassay responses of *Culex quinquefasciatus* and *Culex tarsalis* (Diptera: Culicidae) to chemicals in odors from bermuda grass infusions. J Med Entomol.

[17] Du Y-J, Millar JG (1999). Oviposition responses of gravid *Culex quinquefasciatus* and *Culex tarsalis* to bulrush (Schoenoplectus acutus) infusions. J Am Mosq Control Assoc.

[18] Millar JG, Chaney JD, Mulla MS (1992). Identification of oviposition attractants for *Culex quinquefusciatus* from fermented bermuda grass infusions. J Am Mosq Control Assoc.

[19] Takken W (1999). Chemical signals affecting mosquito behavior. Invertebr Reprod Dev.

[20] Dougherty MJ, Hamilton JGC (1997). Dodecanoic acid is the oviposition pheromone of *Lutzomyia longipalpis*. J Chem Ecol.

[21] World Health Organization. Chagas disease (American trypanosomiasis). Fact sheet N°340. [http://www.who.int/mediacentre/factsheets/fs340/en/index.html].

[22] Schofield CJ (1994). Triatominae. biology & control.

[23] Braga Dias F, Quartier M, Diotaiuti L, Mejía G, Harry H, Lima AC, Davidson R, Mertens F, Lucotte M, Romaña CA (2014). Ecology of Rhodnius robustus Larrousse, 1927 (Hemiptera, Reduviidae, Triatominae) in Attalea palm trees of the Tapajós River Region (Pará State, Brazilian Amazon). Parasites & Vectors.

[24] Lent H, Wygodzinsky P (1979). Revision of the *Triatominae* (Hemiptera, Reduviidae), and their significance as vectors of Chagas’ disease. Bull Am Museum Net Hist.

[25] Schilman PE, Núñez JA, Lazzari CR (1996). Attributes of oviposition substrates affect fecundity in *Rhodnius prolixus*. J Insect Physiol.

[26] Sokal RR, Rohlf FJ (1999). Introduction to Biostatistics.

[27] Zar JH (1999). Biostatistical analysis.

[28] Guha L, Seenivasagan T, Bandyopadhyyay P, Thanvir Iqbal S, Sathe M, Sharma P, et al. Oviposition and flight orientation response of *Aedes aegypti* to certain aromatic aryl hydrazono esters. Parasitol Res. 2012, doi:10.1007/s00436-012-2921-y 10.1007/s00436-012-2921-y 22552771

[29] Tauxe GM, MacWilliam D, Boyle SM, Guda T, Ray A (2013). Targeting a dual detector of skin and CO_2_ to modify mosquito host seeking. Cell.

[30] Logan JG, Birkett MA, Clark SJ, Powers S, Seal NJ, Wadhams LJ, Mordue Luntz AJ, Pickett JA (2008). Identification of human-derived volatile chemicals that interfere with attraction of *Aedes aegypti* mosquitoes. J Chem Ecol.

