# Supplementary figures and images for: Oviposition in the blood-sucking insect Rhodnius prolixus is modulated by host odors
Source: Parasit Vectors. 2015 May 9;8:265. doi: 10.1186/s13071-015-0867-5 (PMC4429358; doi:10.1186/s13071-015-0867-5)

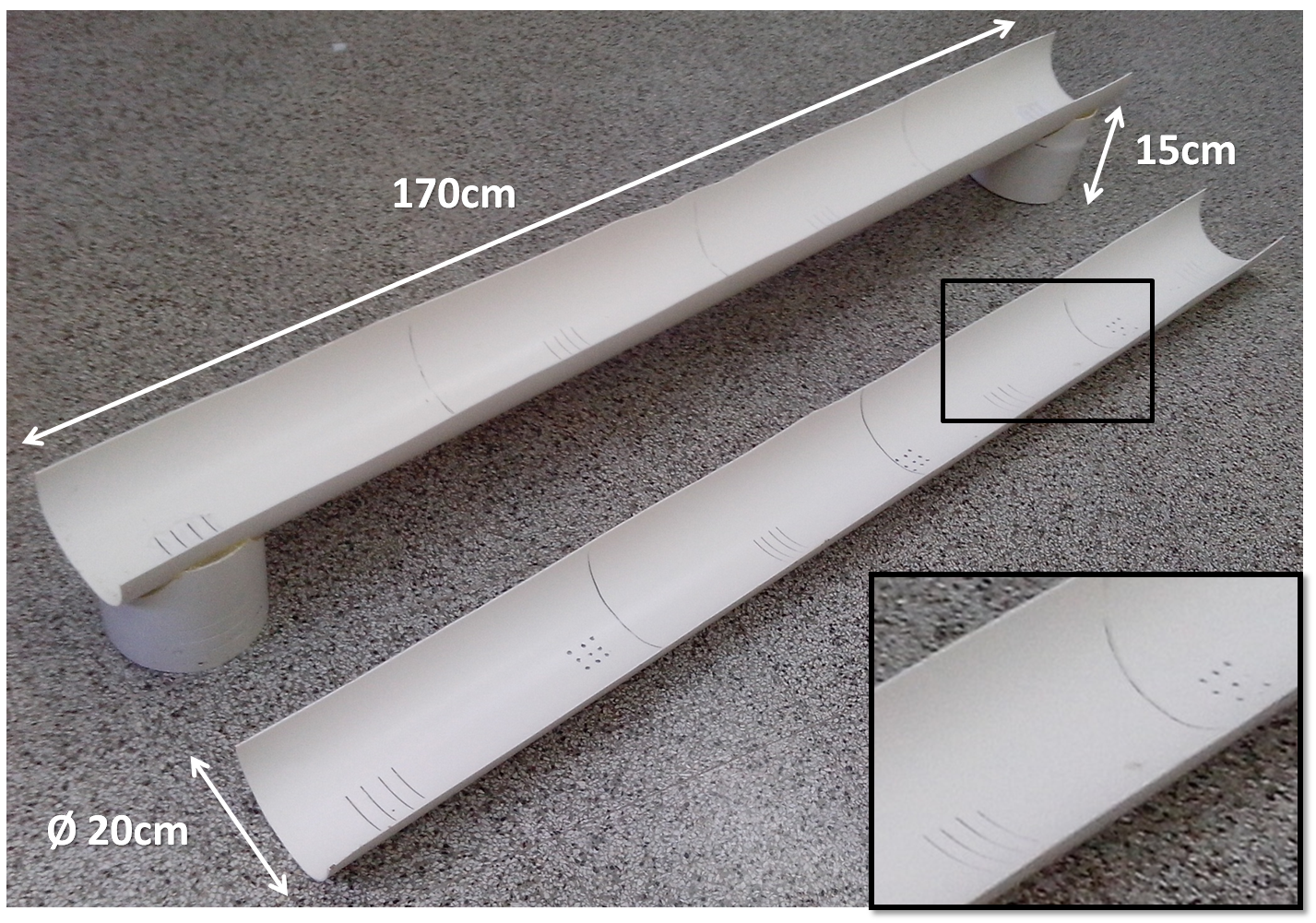

Supplement: Additional file 1: Figure S1. — Superior view of the experimental arena used. The photo shows groups of nine holes and four slits distributed symmetrically along the arena to ensure air circulation. The photo also shows a group of four slits that allowed odor stimuli to reach the arena from the stimuli columns. The inset shows a group of holes and a group of slits distributed symmetrically. [file 13071_2015_867_MOESM1_ESM.tiff]
